# Pre-Treatment Tumor Growth Rate Predicts Clinical Outcomes of Patients With Advanced Non-Small Cell Lung Cancer Undergoing Anti-PD-1/PD-L1 Therapy

**DOI:** 10.3389/fonc.2020.621329

**Published:** 2021-01-19

**Authors:** Li-na He, Xuanye Zhang, Haifeng Li, Tao Chen, Chen Chen, Yixin Zhou, Zuan Lin, Wei Du, Wenfeng Fang, Yunpeng Yang, Yan Huang, Hongyun Zhao, Shaodong Hong, Li Zhang

**Affiliations:** ^1^ State Key Laboratory of Oncology in South China, Guangzhou, China; ^2^ Collaborative Innovation Center for Cancer Medicine, Guangzhou, China; ^3^ Department of Medical Oncology, Sun Yat-sen University Cancer Center, Guangzhou, China; ^4^ Department of Nuclear Medicine, Sun Yat-sen University Cancer Center, Guangzhou, China; ^5^ Department of Radiation Oncology, Sun Yat-sen University Cancer Center, Guangzhou, China; ^6^ Department of VIP Region, Sun Yat-sen University Cancer Center, Guangzhou, China; ^7^ Department of Clinical Research, Sun Yat-sen University Cancer Center, Guangzhou, China

**Keywords:** progression-free survival, non-small cell lung cancer, NSCLC, anti-PD-1/PD-L1 therapy, immunotherapy, tumor growth rate

## Abstract

Tumor growth rate (TGR; percent size change per month [%/m]) is postulated as an early radio-graphic predictor of response to anti-cancer treatment to overcome limitations of RECIST. We aimed to evaluate the predictive value of pre-treatment TGR (TGR_0_) for outcomes of advanced non-small cell lung cancer (aNSCLC) patients treated with anti-PD-1/PD-L1 monotherapy. We retrospectively screened all aNSCLC patients who received PD-1 axis inhibitors in Sun Yat-Sen University Cancer Center between August 2016 and June 2018. TGR_0_ was calculated as the percentage change in tumor size per month (%/m) derived from two computed tomography (CT) scans during a “wash-out” period before the initiation of PD-1 axis inhibition. Final follow-up date was August 28, 2019. The X-tile program was used to identify the cut-off value of TGR_0_ based on maximum progression-free survival (PFS) stratification. Patients were divided into two groups per the selected TGR_0_ cut-off. The primary outcome was the difference of PFS between the two groups. The Kaplan-Meier methods and Cox regression models were performed for survival analysis. A total of 80 eligible patients were included (54 [67.5%] male; median [range] age, 55 [30-74] years). Median (range) TGR_0_ was 21.1 (-33.7-246.0)%/m. The optimal cut-off value of TGR_0_ was 25.3%/m. Patients with high TGR_0_ had shorter median PFS (1.8 months; 95% CI, 1.6 - 2.1 months) than those with low TGR_0_ (2.7 months; 95% CI, 0.5 - 4.9 months) (*P* = 0.005). Multivariate Cox regression analysis revealed that higher TGR_0_ independently predicted inferior PFS (hazard ratio [HR] 1.97; 95% CI, 1.08-3.60; *P* = 0.026). Higher TGR_0_ was also significantly associated with less durable clinical benefit rate (34.8% vs. 8.8%, *P* = 0.007). High pre-treatment TGR was a reliable predictor of inferior PFS and clinical benefit in aNSCLC patients undergoing anti-PD-1/PD-L1 monotherapy. The findings highlight the role of TGR_0_ as an early biomarker to predict benefit from immunotherapy and could allow tailoring patient’s follow-up.

## Introduction

In recent years, immune checkpoint inhibitors (ICIs), including anti-programmed cell death 1 (PD-1) or anti-programmed cell death ligand 1 (PD-L1) therapies, have revolutionized the treatment modalities of advanced non-small cell lung cancer (aNSCLC) (1–5). However, only a small subset of patients have durable response to anti-PD-1/PD-L1 monotherapy and its clinical application was challenged by its atypical response patterns such as hyperprogressive disease (HPD), delayed response, mixed response and pseudoprogressive disease (6, 7). Numerous studies have been conducted to explore early biomarkers to predict response and survival outcomes in patients undergoing ICI treatment (8).

The Response Evaluation Criteria in Solid Tumors (RECIST) criteria provide an objective and standardized response evaluation benchmark for anticancer therapies (9). However, RECIST-based treatment response evaluation does not take into account the tumor growth kinetics (10). Therefore, the RECIST criteria can only be reliably used to compare progression-free survival (PFS) in patients with relatively uniform tumor growth rate when the radiographic evaluation intervals are fixed. Furthermore, RECIST-defined objective response does not always conform the clinical benefit from anticancer treatment (11–15). Also, the RECIST criteria do not provide pre-treatment parameters for earlier prediction of clinical benefit. Thus, it is of clinical relevance to identify other early and inexpensive predictors of benefit from ICI treatment to overcome the limitations of RECIST criteria.

Uncontrolled growth is one of the hallmarks of malignant cells. Fast-growing tumors are associated with the aggressiveness of the tumor, larger tumor bulk, relatively higher sensitivity to cytotoxic agents, significant aberrant neoangiogenesis and altered immune microenvironment (16). Tumor growth rate (TGR) provides quantitative assessment of change in tumor volume over time according to RECIST-defined sum of the longest diameters of the target lesions (SLD) from two computed tomography (CT) scans and time interval between them (17). Previous studies have showed that TGR was correlated with treatment response or clinical outcomes in patients with neuroendocrine carcinoma, renal cell carcinoma, or hepatocellular carcinoma treated with angiogenesis inhibitors or transarterial chemoembolization (18–23). These findings suggested that TGR could serve as an early radiological biomarker to predict patient’s survival outcomes and to tailor radiological follow-up strategies and patients’ management.

To our knowledge, no previous studies had illustrated the association of pre-treatment TGR with clinical outcomes of aNSCLC patients treated with ICI. Considering that the natural tumor growth kinetics might significantly impact the tumor microenvironment, we hypothesized that pre-treatment TGR could predict PFS of aNSCLC patients undergoing anti-PD-1/PD-L1 immunotherapy.

## Methods

### Data Source

**Figure 1:**
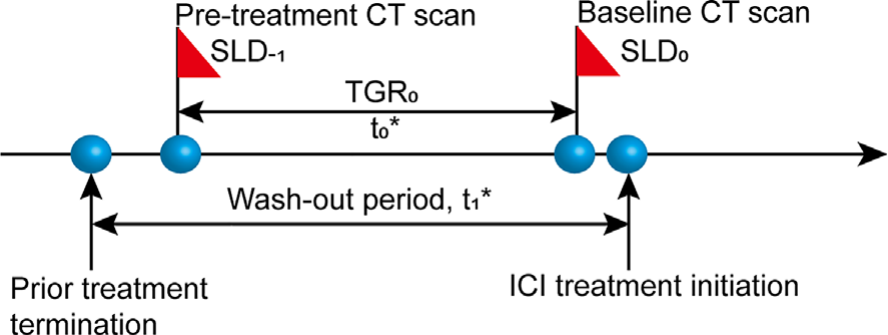
Diagram of computed tomography (CT) scan timepoints. SLD_-1_, sum of the longest diameters of the target lesions at pre-treatment CT scan; SLD_0_, sum of the longest diameters of the target lesions at baseline CT scan; t_0_, time interval between pre-treatment and baseline CT scans, 2 weeks≤ t_0_ ≤ 3 months; t_1,_ wash-out period before the initiation of ICI treatment without any anti-cancer treatment.

We conducted a retrospective review of electronic medical records from all aNSCLC patients undergoing ICI therapy (N = 172) at Sun Yat-Sen University Cancer Center (SYSUCC) between August 2016 and June 2018. Eligible patients should have two consecutive computed tomography (CT) scans before the initiation of ICI treatment (termed “wash-out period”) and receiving no anti-cancer treatment between the two scans (**Figure 1**). Exclusion criteria were as follows: lacking available pre-treatment CT scan; time interval between pre-treatment (defined as the time prior to baseline) and baseline (defined as the time of ICI initiation) CT scans shorter than 2 weeks or longer than 3 months (tumor growth kinetics should be assessed during a proper period) (24); lacking measurable lesions by RECIST version 1.1 (RECIST 1.1) at baseline CT scans; having received local anti-cancer therapy such as radiotherapy and radiofrequency ablation during ICI treatment or follow-up. The study was approved by the Institutional Review Board of SYSUCC and written informed consent was waived due to the retrospective nature of the study. Our study followed the Strengthening the Reporting of Observational Studies in Epidemiology (STROBE) reporting guideline (25).

TGR is expressed as the percentage change in tumor size per month (%/m) and calculated based on a published formula (17, 21): TGR = 100 * [exp (TG) – 1]; TG= (3 * log(D_2_/D_1_))/t, where t = (date_2_ – date_1_ + 1)/30.44, indicating the time interval in months between two CT imaging evaluations, and TG is the growth rate. Tumor size (D) derives from the sum of the longest diameters (SLD) of the target lesions according to RECIST 1.1. D_1_ = tumor size at date_1_; D_2_ = tumor size at date_2_. We simplified the formula into this form: TGR = 100 * ((D_2_/D_1_)^1.303/t^ – 1).

For all patients, we collected data including demographic characteristics, clinical and radiological information: sex, age; previous lines of systemic therapies, smoking status, histology, clinical stage, Eastern Cooperative Oncology Group (ECOG) performance status (PS), alterations in driver genes including epidermal growth factor receptor (EGFR) and anaplastic lymphoma kinase (ALK); date of CT scans, SLD, status of non-measurable lesions and new lesions. The same assessment method and same technique (CT) were used at each imaging assessment point. For patients had disease progression with new lesions, tumor size was determined by target lesions only, while the occurrence of new lesions was recorded. In case of multiple alternative pre-baseline images, we selected the latest one to baseline for analysis. Missing data were recorded as not available.

### Response and Endpoint Evaluation

All response and outcome evaluation were determined as per RECIST 1.1 by two senior radiologists blinded to patients’ information. Discrepancy was solved by consensus. Follow-up CT scans were performed according to the physicians’ discretion without predetermined intervals. Patients underwent tumor assessment until immunotherapy termination due to any reasons. The primary endpoint was PFS, defined as time from ICI initiation to radiologically-defined progression or death from any causes. The secondary endpoints were durable clinical benefit (DCB) rate, overall response rate (ORR) and overall survival (OS). DCB was defined as achieving any one of complete response (CR), partial response (PR) or stable disease (SD) that lasted for at least 6 months from baseline. The data cut-off date was August 28, 2019.

### Statistical Analysis

We used the X-tile program (Yale University School of Medicine, New Haven, CT, USA) to determine the optimal cut-off values of TGR_0_ and baseline SLD (SLD_0_) to maximize PFS differentiation (26). According to the TGR_0_ cut-off point, patients were divided into two groups, and baseline characteristics between the two groups were compared. Continuous variables were expressed as median (range) and analyzed using Mann-Whitney U-test or independent t-test depending on the normality of distribution; categorical variables were expressed as number (%) and analyzed using Fisher’s exact test or Chi-square test as appropriate. PFS and OS survival curves were generated using Kaplan-Meier method and the differences were compared using the log-rank test. Investigation of the effect of TGR_0_ and other baseline parameters on treatment outcomes was performed using univariate and multivariate Cox regression analyses. Two-sided *P*-value of less than 0.05 was considered statistically significant. All statistical analyses were performed using the R software version 3.6.1 (https://www.r-project.org/).

## Results

### Patient Characteristics

**Figure 2:**
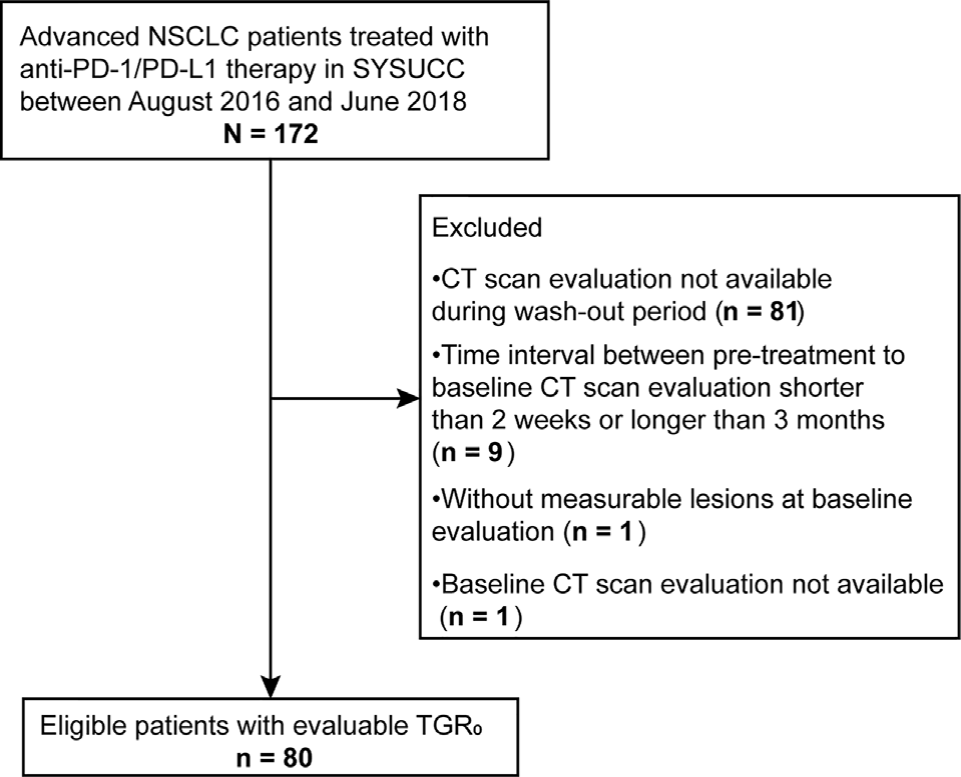
Flowchart of patient screening process.

Of the 172 patients screened, 80 met the eligible criteria (**Figure 2**). The median follow-up time was 23.6 months (95% confidence interval [CI], 13.5 - 33.7 months). Baseline characteristics of all patients were depicted in **Table 1**. The median (range) age was 55 (30 - 74) years. 54 (67.5%) were male, 48 (60.0%) were non-smokers, 31 (38.7%) had squamous histology, 19 (23.8%) had previously received radiotherapy and 36 (45.0%) had three or more metastatic sites. 31 patients (38.7%) had an ECOG PS of 0, with 4 (5.0%) patients scoring at 2 or 3. The median (range) duration between pre-treatment and baseline CT scans was 1.0 (0.5 - 3.0) months. The median (range) SLD_0_ was 74 (17 - 231) mm, and the median (range) TGR_0_ was 21.1(-33.7 - 246.0) %/m. At data cut-off, 42 out of 80 (53.5%) patients died.

**Table 1:** Patient characteristics at baseline (n = 80).

Patient characteristics	No. (%)
**Age, years**	
Median (range)	55 (30-74)
< 55	40 (50.0)
≥ 55	40 (50.0)
**Gender**	
Male	54 (67.5)
Female	26 (32.5)
**ECOG PS**	
0	31 (38.7)
1	45 (56.3)
2-3	4 (5.0)
**Smoking status**	
Never smoker	48 (60.0)
Current or former smoker	32 (40.0)
**Histology**	
Squamous cell carcinoma	31 (38.7)
Nonsquamous cell carcinoma	49 (61.3)
**No. of prior treatment lines**	
0-1	49 (61.3)
≥2	31 (38.7)
**No. of metastatic sites**	
1-2	44 (55.0)
≥3	36 (45.0)
**Prior radiotherapy**	
Yes	19 (23.8)
No	61 (76.2)
**Type of ICI**	
Pembrolizumab	34 (42.5)
Atezolizumab	7 (8.7)
Nivolumab	18 (22.5)
Camrelizumab	21 (26.3)
**EGFR mutation status**	
Positive	10 (12.5)
Negative	51 (63.7)
Not available	19 (23.8)
**ALK translocation**	
Positive	4 (5.0)
Negative	52 (65.0)
Not available	24 (30.0)
**SLD_0_, mm**	
Median (range)	74 (17-231)
≤ 130	70 (87.5)
> 130	10 (12.5)
**t_0_, months**	
Median (range)	1.0 (0.5-3.0)
**TGR_0_, %/m**	
Median (range)	21.1 (-33.7-246.0)
≤ 25.3	46 (57.5)
> 25.3	34 (42.5)
**RECIST response**	
PR	10 (12.5)
SD	19 (23.7)
PD	46 (57.5)
NE	5 (6.3)

ECOG PS, Eastern Cooperative Oncology Group performance status; ICI, immune checkpoint inhibitor; EGFR, epidermal growth factor receptor; ALK, anaplastic lymphoma kinase; SLD_0_, sum of the longest diameters of the target lesions at baseline; t_0_, time interval from pre-treatment to baseline CT evaluation; TGR_0_, pre-treatment tumor growth rate; RECIST, Response Evaluation Criteria in Solid Tumors; PR, partial response; SD, stable disease; PD, progressive disease, NE, not evaluable.

As per RECIST 1.1, 10 (12.5%) patients achieved PR as best overall response, 19 (23.7%) achieved SD, 46 (57.5%) had PD and 5 (6.3%) had non-evaluable response. Overall response rate (ORR) was 12.5%, and DCB rate was 23.8%. The median PFS and OS were 2.1 months (95% CI, 1.8 - 3.1 months) and 23.6 months (95% CI, 14.8 - not reached months), respectively.

### Cut-Off Points by X-Tile Program

**Figure 3:**
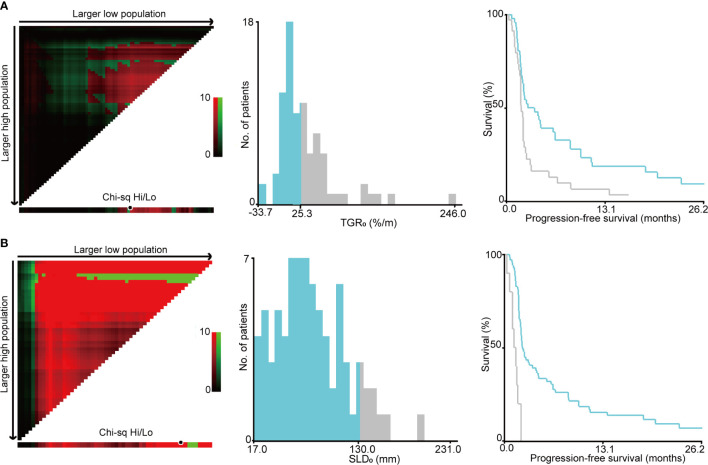
Identification of cut-off values based on progression-free survival by X-tile analysis. **(A)** The optimal cut-off value for sum of the longest diameters of target lesions at baseline (SLD_0_) was 130 mm (χ^2^ = 22.995, *P* < 0.001). **(B)** The optimal cut-off value for pre-treatment tumor growth rate (TGR_0_) based on PFS was 25.3%/m (χ^2^ = 7.546, *P* = 0.112).

The optimal cut-off points of SLD_0_ and TGR_0_ based on PFS separation were 130 mm (χ^2^ = 22.995, *P* < 0.001) and 25.3%/m (χ^2^ = 7.546, *P* = 0.112), respectively (**Figure 3**). Both cut-off points showed the maximum prognostic effects in predicting PFS. According to the TGR_0_ cut-off point, we divided patients into two groups: low group, TGR_0_ ≤ 25.3%/m (n = 46); high group, TGR_0_ > 25.3%/m (n = 34). The clinicopathological characteristics of TGR_0_ strata were shown in **Table 2**. Low TGR_0_ was significantly associated with positive smoking history (*P* = 0.034) and RECIST-defined best response (*P* = 0.028). There was no significant association between TGR_0_ and other factors including age, gender, ECOG PS, histology, number of prior therapy lines, number of metastatic sites, history of prior radiotherapy, EGFR and ALK status (all with *P* > 0.05).

**Table 2:** Association of TGR_0_ with other parameters (n = 80).

	TGR_0_ ≤ 25.3%/m (n=46) No. (%)	TGR_0_ > 25.3%/m (n=34) No. (%)	P-value
**Age**			0.873
Median (range)	56 (33-77)	52 (30-74)	
**Gender**			0.056
Male	35 (64.8)	19 (35.2)	
Female	11 (42.3)	15 (57.7)	
**ECOG PS**			0.932
0	17 (54.8)	14 (45.2)	
1	27 (60.0)	18 (40.0)	
2-3	2 (50.0)	2 (50.0)	
**Smoking status**			0.034
Never smoker	23 (47.9)	25 (52.1)	
Current or former smoker	23 (71.9)	9 (28.1)	
**Histology**			0.935
Squamous cell carcinoma	18 (58.1)	13 (41.9)	
Nonsquamous cell carcinoma	28 (57.1)	21 (42.9)	
**No. of prior treatment lines**			0.935
0-1	28 (57.1)	21 (42.9)	
≥2	18 (58.1)	13 (41.9)	
**No. of metastatic sites**			0.220
1-2	28 (63.6)	16 (36.4)	
≥3	18 (50.0)	18 (50.0)	
**Prior radiotherapy**			0.623
Yes	10 (52.6)	9 (47.4)	
No	36 (59.0)	25 (41.0)	
**EGFR mutation status**			0.983
Positive	6 (60.0)	4 (40.0)	
Negative	29 (56.9)	22 (43.1)	
Not available	11 (57.9)	8 (42.1)	
**ALK translocation**			0.390
Positive	1 (25.0)	3 (75.0)	
Negative	32 (61.5)	20 (38.5)	
Not available	13 (54.2)	11 (45.8)	
**SLD_0_, mm**			0.368
Median (range)	72 (17-158)	76 (19-231)	
**RECIST response**			0.028
PR	7 (70.0)	3 (30.0)	
SD	15 (78.9)	4 (21.1)	
PD	20 (43.5)	26 (56.5)	
NE	4 (80.0)	1 (20.0)	

ECOG PS, Eastern Cooperative Oncology Group performance status; EGFR, epidermal growth factor receptor; ALK, anaplastic lymphoma kinase; SLD_0_, sum of the longest diameters of the target lesions at baseline; RECIST, Response Evaluation Criteria in Solid Tumors; PR, partial response; SD, stable disease; PD, progressive disease; NE, not evaluable.

### Association of TGR_0_ With Clinical Outcomes

**Figure 4:**
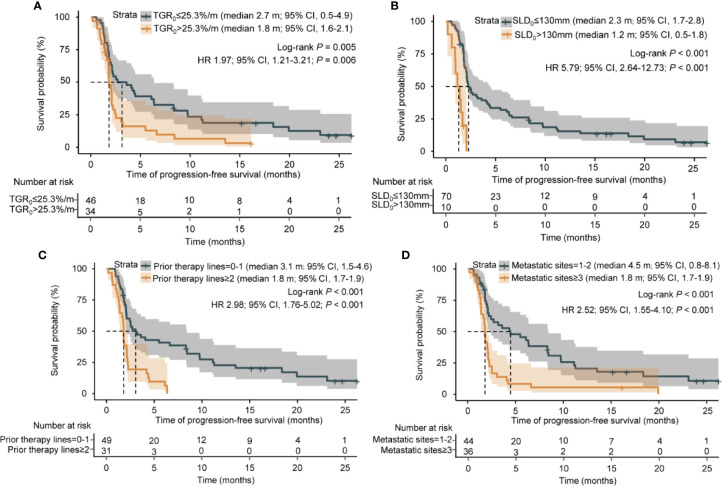
Kaplan-Meier analysis of progression-free survival. **(A)** Progression-free survival by pre-treatment tumor growth rate. **(B)** Progression-free survival by sum of the longest diameters of the target lesions at baseline. **(C)** Progression-free survival by number of prior therapy lines. **(D)** Progression-free survival by number of metastatic sites.

Kaplan-Meier survival analyses revealed that patients with high TGR_0_ experienced inferior median PFS (1.8 months; 95% CI, 1.6 - 2.1 months) compared with those with low TGR_0_ (2.7 months; 95% CI, 0.5 - 4.9 months) (log-rank *P* = 0.005) (**Figure 4A**). The 12-month PFS rate was 5.9% vs. 17.4% in patients with high vs. low TGR_0._ Univariate analyses revealed that the following factors were significantly associated with inferior PFS: higher TGR_0_ (hazard ratio [HR] 1.97; 95% CI, 1.21 - 3.21; *P* = 0.006), larger SLD_0_ (HR 5.79, 95% CI, 2.64 - 12.73; *P* < 0.001), two or more lines of prior therapy for advanced disease (HR 2.98; 95% CI, 1.76 - 5.02; *P* < 0.001), three or more metastatic sites (HR 2.52; 95% CI, 1.55 - 4.10; *P* < 0.001), ECOG PS of 2 to 3 (HR 3.35; 95% CI, 1.14 - 9.80; *P* = 0.027) and ALK rearrangement (HR 4.69; 95% CI, 1.61 - 13.70; *P* = 0.005) (**Figures 4A–D**, **Table 3**). Patients with EGFR mutant tumor also exhibited shorter PFS, with borderline significance (HR 2.00; 95% CI, 0.98 - 4.06; *P* = 0.056). In multivariate Cox model included all analyzed factors in univariate analyses, we found that higher TGR_0_ (HR 1.97; 95% CI, 1.08 - 3.60; *P* = 0.026), larger SLD_0_ (HR 10.70; 95% CI, 4.20 - 27.23; *P* < 0.001) and two or more lines of prior therapy (HR 3.36; 95% CI, 1.58 - 7.15; *P* = 0.002) remained significantly associated with shorter PFS (**Table 3**). Negative history of prior radiotherapy (HR 1.92; 95% CI, 0.96 - 3.83; *P* = 0.066) and three or more metastatic sites (HR 1.97; 95% CI, 1.00 - 3.88; *P* = 0.051) also tended to predict inferior PFS (**Table 3**).

**Table 3:** Univariate and multivariate analyses of progression-free survival.

	Univariate analysis		Multivariate analysis	
	HR (95% CI)	*P-*value	HR (95% CI)	*P-*value
**Age, years**				
< 55	1.40 (0.88-2.24)	0.156	1.46 (0.80-2.67)	0.212
≥ 55	1 [Reference]	NA	1 [Reference]	NA
**Gender**				
Male	1 [Reference]	NA	1 [Reference]	NA
Female	1.49 (0.91-2.45)	0.115	1.44 (0.74-2.79)	0.282
**ECOG PS**				
0	1 [Reference]	NA	1 [Reference]	NA
1	1.09 (0.67-1.78)	0.721	0.99 (0.56-1.75)	0.979
2-3	3.35 (1.14-9.80)	0.027	1.49 (0.39-5.79)	0.561
**Smoking status**				
Never smoker	1.16 (0.72-1.86)	0.545	1 [Reference]	NA
Current or former smoker	1 [Reference]	NA	1.18 (0.60-2.33)	0.637
**Histology**				
Squamous cell carcinoma	1 [Reference]	NA	1 [Reference]	NA
Nonsquamous cell carcinoma	1.14 (0.70-1.87)	0.590	1.11 (0.55-2.24)	0.780
**No. of prior treatment lines**				
0-1	1 [Reference]	NA	1 [Reference]	NA
≥2	2.98 (1.76-5.02)	<0.001	3.36 (1.58-7.15)	0.002
**No. of metastatic sites**				
1-2	1 [Reference]	NA	1 [Reference]	NA
≥3	2.52 (1.55-4.10)	<0.001	1.97 (1.00-3.88)	0.051
**Prior radiotherapy**				
Yes	1.10 (0.64-1.90)	0.736	1 [Reference]	NA
No	1 [Reference]	NA	1.92 (0.96-3.83)	0.066
**EGFR mutation status**				
Negative	1 [Reference]	NA	1 [Reference]	NA
Positive	2.00 (0.98-4.06)	0.056	1.00 (0.39-2.61)	0.917
Not available	1.41 (0.80-2.49)	0.233	2.80 (0.73-10.77)	0.135
**ALK translocation**				
Negative	1 [Reference]	NA	1 [Reference]	NA
Positive	4.69 (1.61-13.70)	0.005	2.43 (0.67-8.82)	0.177
Not available	1.24 (0.74-2.08)	0.424	0.59 (0.18-1.86)	0.365
**SLD_0_, mm**				
≤ 130	1 [Reference]	NA	1 [Reference]	NA
> 130	5.79 (2.64-12.73)	<0.001	10.70 (4.20-27.23)	<0.001
**TGR_0_, %/m**				
≤ 25.3	1 [Reference]	NA	1 [Reference]	NA
> 25.3	1.97 (1.21-3.21)	0.006	1.97 (1.08-3.60)	0.026

HR, hazard ratio; CI, confidence interval; NA, not applicable; ECOG PS, Eastern Cooperative Oncology Group performance status; EGFR, epidermal growth factor receptor; ALK, anaplastic lymphoma kinase; SLD_0_, sum of the longest diameters of the target lesions at baseline; TGR_0_, pre-treatment tumor growth rate.

**Figure 5:**
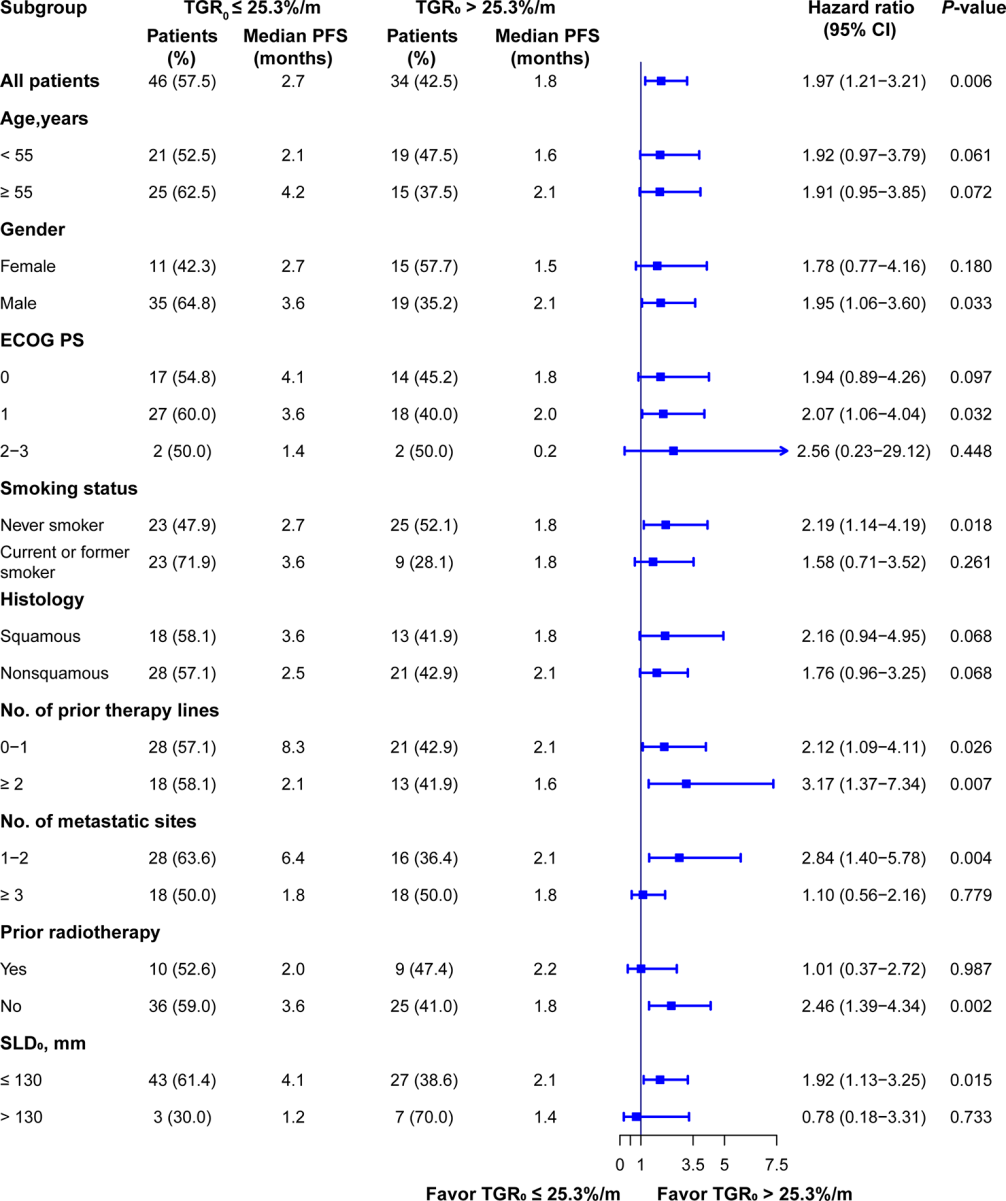
Subgroup analysis of PFS according to TGR_0_ stratification. Findings were examined by Cox proportional hazard regression analysis.

To further validate the effect of TGR_0_ on PFS, we performed subgroup analysis based on specific baseline parameters. TGR_0_ predicted efficacy of ICI in NSCLC patients across almost all the subgroups including age, ECOG PS of 0 or 1, male, never smoker, histology, prior treatment lines, 1 or 2 metastatic sites, negative history of prior radiotherapy and small SLD_0_ (**Figure 5**). In the histology subgroup, 43 were histologically conformed lung adenocarcinomas. Among them, 25 had low TGR_0_ level, with 18 grouped into high TGR_0_ strata. High TGR_0_ also tended to predicted shorter PFS (HR 1.75; 95% CI, 0.91 - 3.37), though not statistically significant (*P* = 0.097). However, in patients with metastatic sites of ≥ 3, ECOG PS of 2 to 3, positive history of prior radiotherapy and those with large SLD_0,_ TGR_0_ did not have impact on PFS.

TGR_0_ did not have impact on OS (HR 1.24; 95% CI, 0.64-2.39; log-rank *P* = 0.519) (**Figure S1**). In multivariate Cox regression analysis, ECOG PS of 1 (HR 2.65; 95% CI 1.14 - 6.19; *P* = 0.024), ECOG PS of 2 to 3 (HR 30.62; 95% CI 3.61 - 260.01; *P* = 0.002), two or more prior treatment lines (HR 2.65; 95% CI 1.14 - 6.16; *P* = 0.024), without EGFR mutation (HR 7.12; 95% CI 1.32 - 38.53; *P* = 0.023), larger SLD_0_ (HR 8.24; 95% CI 2.84 - 23.85; *P* < 0.001) were significantly associated with poorer OS (**Table S1**). Patients with low TGR_0_ achieved significantly higher DCB rate compared with those with high TGR_0_ (16 of 46 [34.8%] vs. 3 of 34 [8.8%], *P* = 0.007). However, there was only a trend towards increased ORR in patients with low TGR_0_ (7 of 46 [15.2%] vs. 3 of 34 [8.8%]; *P* = 0.505).

## Discussion

Early prediction of response to anti-cancer therapy is important for selecting patients that are more likely to benefit from such treatment and optimizing radiological follow-up strategies. The results from our study suggested that higher pre-treatment tumor growth rate (TGR_0_) played a role in predicting inferior PFS for aNSCLC patients treated with anti-PD-1/PD-L1 monotherapy. Patients with higher TGR_0_ was also significantly associated with less durable clinical benefit.

Our findings resonated with some previous studies. A *post hoc* analysis from a phase II study revealed that higher pre-treatment TGR tended to be associated with shorter PFS in grade 1 or 2 gastroenteropancreatic neuroendocrine tumors (GEP-NETs) receiving lanreotide (27), and the CLARINET study further validated this finding (18). A TGR_0_ < 4%/m predicted inferior PFS in G1 or G2 NET patients regardless of treatment modalities (21). Similarly, patients with higher pre-treatment tumor growth rate——measured as specific growth rate (SGR) experienced worse PFS in locally advanced NSCLC undergoing definitive chemoradiation therapy (CRT) (28). It was postulated that tumor growth rate may be more biologically and clinically relevant for predicting patient’s clinical outcomes than the RECIST did. The GREPONET study found that TGR_3m_ provide more useful information in predicting patients’ outcomes and had less variability than RECIST_3m_ (21). Another study enrolling 58 aNSCLC patients showed that the deceleration in TGR at first follow-up after the start of ICI therapy was significantly associated with superior OS (29). It is worthy of note that the median (range) of TGR_0_ from these 58 aNSCLC patients and our cohort was comparable (28.0 [−48.6 to 293.7]%/m vs. 21.1 [-33.7-246.0]%/m), indicating the repeatability of the calculation of TGR. Taken together, these results imply that translation of TGR into clinical practice may allow earlier and more precise prediction of clinical outcomes in oncotherapy. Our study further and for the first time showed that the natural tumor growth kinetics, estimated as TGR_0_, could predict the efficacy of ICI in NSCLC. This association was consistent across different subgroups and was maintained in multivariate regression analysis.

The TGR_0_ could therefore have a potential in tailoring on-treatment imaging schemes and early prediction of risk of disease progression. Based on our findings, patients with high TGR_0_ should undergo more frequent follow-up imaging because of their shorter PFS, namely higher risk of experiencing early disease progression; while patients with low TGR_0_ are more likely to have durable clinical benefit and could receive follow-up imaging assessment of longer interval to cut down the radiation exposure and examination cost. TGR also played a role in examining anti-tumor drug activity and guiding “go/no go” decision making in the early drug development. Although the rationale behind the negative impact of TGR_0_ on the efficacy of ICI is unclear, it could be hypothesized that the immune microenviroment of fast-growing tumor is unfavorable for the action of PD-1 axis inhibitors. Another possible explanation is that the time for the adaptive immune response and tumor killing after PD-1 axis inhibition is too long compare with the tumor growth rate. These results implied that fast-growing tumors should avoid being treated with single agent ICI. This could be viewed from the case of small cell lung cancer, which is a typical type of fast-growing tumor and have poor responsiveness to single agent ICI but demonstrates improved survival with chemoimmunotherapy combinations (30). Also, our results highlight the need for future exploration of combining TGR and RECIST criteria to refine the follow-up schemes as well as the role of ICI plus chemotherapy in tumors with high TGR_0_.

Our study failed to observe significant difference in OS between TGR_0_ strata, which might be due to the divergent sensitivity of subsequent treatment (chemotherapy as the mainstream one) in these two groups, the imbalance of subsequent treatment, and the relatively small sample size. Nevertheless, we also found that high TGR_0_ was correlated with low DCB rate and a tendency towards lower ORR. Similar to our observation, Yvonne Purcell et al. elucidated that the mean pre-treatment TGR was not significant different between the objective response (OR) and non-OR group in hepatocellular carcinoma treated with transarterial chemoembolization (19). This is clinically relevant because it has been showed that ORR was poorly correlated with long-term survival for immunotherapy (12, 31), indicating the inadequacy of RECIST criteria which only capture tumor volume change but miss out temporal information. Taken together, these results indicate that TGR_0_ is a predictive rather than a prognostic factor for aNSCLC patients undergoing ICI therapy. It is therefore more reliable to guide patients’ management in clinical practice.

Our study has several limitations. First, the study was retrospectively conducted at a single institute with a moderate sample size. Statistical power was limited and could explain why the ORR and the OS between high and low TGR_0_ groups did not reach statistical significance. Prospective or external validation of our work is required in another cohort with larger sample size. However, we think our finding is relatively reliable and reproducible since the predictive value of TGR is confirmed in various cancers undergoing different treatment therapies. Second, the strict inclusion criteria, especially the requirements of two consecutive imaging during wash-out period, may cause potential selection bias. Third, target lesions selected for calculation of TGR might not represent the whole tumor burden as new lesions and non-target lesions were not taken into account. Dissociated or mixed response phenomenon may confound the accurate tumor kinetics assessment after treatment initiation (32). Fourth, Limited to the retrospective nature of our work, we were unable to estimate predictive value of PD-L1 status and TMB level for only 3 patients had detected these two items. The main reason was that PD-L1 and TMB testing were not mandatory for using immunotherapy regimens since most patients in our study received ICIs in two or more lines of treatment. When we sought to re-evaluate PD-L1 and TMB status, the tissues were insufficient because all were from small biopsies. Last, the clinical application of TGR_0_ may be limited by the economic and ethical consideration of additional imaging evaluation required during wash-out period. However, considering the risk of early disease progression and cost of ICI treatments, we think such procedure might still have clinical relevance. Despite these limitations, our findings suggested that TGR_0_ has potential value for clinical utility by predicting risk of progression and providing complementary information to RECIST criteria.

## Conclusions

Higher pre-treatment TGR was significantly associated with inferior PFS and less durable clinical benefit in aNSCLC patients undergoing anti-PD-1/PD-L1 monotherapy. TGR_0_ could provide additional information for predicting the efficacy of immune checkpoint inhibition and facilitate tailoring patient’s management. The potential role of TGR_0_ in the treatment decision requires further validation in another cohort and future prospective studies.

## Data Availability Statement

All the data supporting the findings of this study are available from the corresponding authors upon reasonable request.

## Ethics Statement

The studies involving human participants were reviewed and approved by the Institutional Review Board of Sun Yat-sen University Cancer Center. Written informed consent for participation was not required for this study in accordance with the national legislation and the institutional requirements.

## Author Contributions

SH and LZ put forward the study concept and design of the work. All authors contributed to the acquisition, analysis, or interpretation of data. L-NH, XZ, and HL drafted the manuscript. SH and LZ helped with funding acquisition. SH and YH edited the manuscript. LZ contributed to the study supervision. All authors contributed to the article and approved the submitted version.

## Funding

This study was funded by grants 81972898 and 81872499 from the National Natural Science Funds of China; 2019A1515011090 from the Natural Science Funds of Guangdong Province; the 16zxyc04 from the Outstanding Young Talents Program of Sun Yat-sen University Cancer Center; The funding sources had no role in the design and conduct of the study; collection, management, analysis, and interpretation of the data; preparation, review, or approval of the manuscript; and decision to submit the manuscript for publication.

## Conflict of Interest

The authors declare that the research was conducted in the absence of any commercial or financial relationships that could be construed as a potential conflict of interest.

## Supplementary Material

Supplementary Figure 1 Kaplan-Meier analysis of overall survival by pre-treatment tumor growth rate.
