# Supplementary figures and images for: Pre-Treatment Tumor Growth Rate Predicts Clinical Outcomes of Patients With Advanced Non-Small Cell Lung Cancer Undergoing Anti-PD-1/PD-L1 Therapy
Source: Front Oncol. 2021 Jan 19;10:621329. doi: 10.3389/fonc.2020.621329 (PMC7863973; doi:10.3389/fonc.2020.621329)

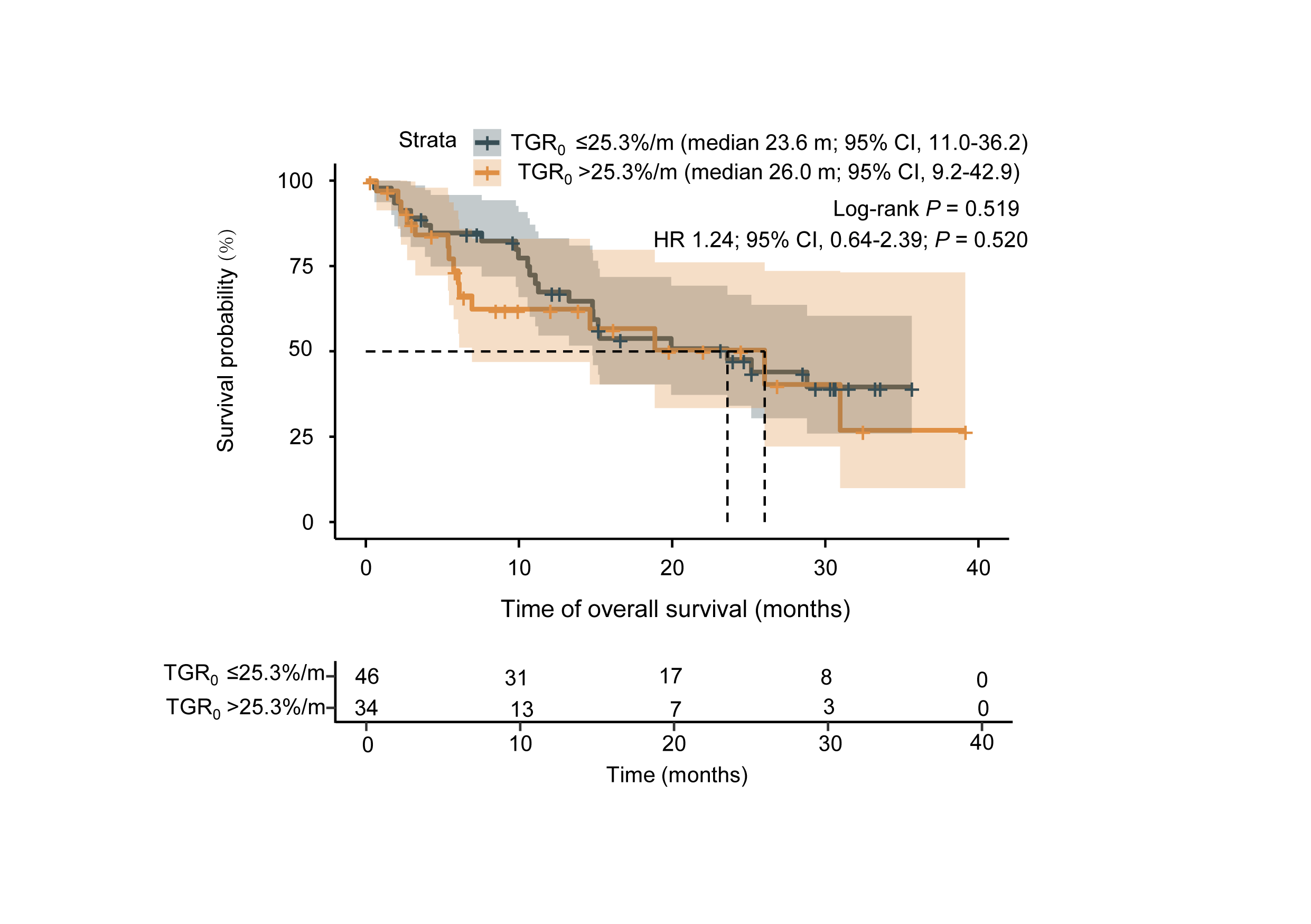

Supplement: Supplementary Figure 1 — Kaplan-Meier analysis of overall survival by pre-treatment tumor growth rate. [file Image_1.tif]
