# Supplementary material for: Pre-Treatment Tumor Growth Rate Predicts Clinical Outcomes of Patients With Advanced Non-Small Cell Lung Cancer Undergoing Anti-PD-1/PD-L1 Therapy
Source: Front Oncol. 2021 Jan 19;10:621329. doi: 10.3389/fonc.2020.621329 (PMC7863973; doi:10.3389/fonc.2020.621329)
Supplement: Supplementary file 2 [file DataSheet_1.pdf]

Table S1. Univariate and multivariate analyses of prognostic factors for overall survival

	Univariate analysis		Multivariate analysis	
	**HR (95% CI)**	***P*-value**	**HR (95% CI)**	***P*-value**
**Age, years**				
< 55	1 [Reference]	NA	1 [Reference]	NA
≥ 55	1.27 (0.67-2.41)	0.461	1.68 (0.77-3.67)	0.190
**Gender**				
Male	1.06 (0.53-2.11)	0.864	1 [Reference]	NA
Female	1 [Reference]	NA	1.30 (0.52-3.25)	0.581
**ECOG PS**				
0	1 [Reference]	NA	1 [Reference]	NA
1	2.12 (1.02-4.39)	0.044	2.65 (1.14-6.19)	0.024
2-3	10.47 (2.07-53.01)	0.005	30.62 (3.61-260.01)	0.002
**Smoking status**				
Never smoker	1.10 (0.57-2.10)	0.782	1.02 (0.40-2.61)	0.969
Current or former smoker	1 [Reference]	NA	1 [Reference]	NA
**Histology**				
Squamous cell carcinoma	1.91 (0.99-3.68)	NA	1 [Reference]	NA
Nonsquamous cell carcinoma	1 [Reference]	0.053	1.17 (0.44-3.13)	0.749
**No. of prior treatment lines**				
0-1	1 [Reference]	NA	1 [Reference]	NA
≥2	1.71 (0.90-3.25)	0.102	2.65 (1.14-6.16)	0.024
**No. of metastatic sites**				
1-2	1 [Reference]	NA	1 [Reference]	NA
≥3	1.21 (0.63-2.29)	0.568	1.38 (0.56-3.37)	0.484
**Prior radiotherapy**				
Yes	1.81 (0.87-3.79)	0.114	1.71 (0.74-3.94)	0.208
No	1 [Reference]	NA	1 [Reference]	NA
**EGFR mutation status**				
Positive	1 [Reference]	NA	1 [Reference]	NA
Negative	2.43 (0.57-10.30)	0.229	7.12 (1.32-38.53)	0.023
Not available	4.85 (1.06-22.17)	0.042	843.20 (2.60-717.61)	0.009
**ALK translocation**				
Negative	1 [Reference]	NA	2.12 (0.37-12.22)	0.399
Positive	1.16 (0.27-4.93)	0.842	1 [Reference]	NA
Not available	1.74 (0.87-3.50)	0.119	0.53 (0.03-8.53)	0.651
**SLD_0, mm**				
≤ 130	1 [Reference]	NA	1 [Reference]	NA
> 130	4.90 (2.17-11.06)	< 0.001	8.24 (2.84-23.85)	< 0.001
**TGR_0, %/m**				
≤ 25.3	1 [Reference]	NA	1.00 (0.45-2.26)	0.996
> 25.3	1.24 (0.64-2.39)	0.520	1 [Reference]	NA

Abbreviations: HR, hazard ratio; CI, confidence interval; NA, not applicable; ECOG PS, Eastern Cooperative Oncology Group performance status; EGFR, epidermal growth factor receptor; ALK, anaplastic lymphoma kinase; SLD_0, sum of the longest diameters of the target lesions at baseline; TGR_0, pre-treatment tumor growth rate.
